# Unlocking the potential of up-conversion charging for rapid and high-resolution optical storage with phosphors

**DOI:** 10.1038/s41377-025-01746-9

**Published:** 2025-03-04

**Authors:** Lu Chen, Xueqing Liu, Feng Liu, Chuan Liao, Liangliang Zhang, Jiahua Zhang, Xiao-jun Wang, Yichun Liu

**Affiliations:** 1 Key Laboratory for UV-Emitting Materials and Technology of Ministry of Education, Northeast Normal University, 130024 Changchun, China; 2 State Key Laboratory of Luminescence and Applications, Changchun Institute of Optics, Fine Mechanics and Physics, Chinese Academy of Sciences, 130023 Changchun, China; 3 Department of Physics, Georgia Southern University, Statesboro, GA 30460 USA

**Keywords:** Optical data storage, Optical materials and structures

## Abstract

Current optical storage technologies utilizing phosphor media face challenges in achieving rapid and precise data recording with visible or infrared light, primarily due to the constraints of traditional charging techniques. Here, we introduce a cutting-edge method termed up-conversion charging (UCC) to address these challenges, enabling rapid and high-resolution data storage in phosphors. Our study focuses on the unique two-step ionization and non-linear charging characteristics of UCC in storage phosphors, specifically in a gallate composition Gd_3_Ga_5_O_12_:Cr^3+^. Remarkably, this technique enables data writing with high solution, requiring only 0.01 s of exposure per bit when utilizing a portable laser engraver equipped with visible-emitting diode lasers. The present strategy not only enhances recording efficiency but also ensures long-term data retention and superior rewritability. Moreover, we illustrate the versatility of UCC storage across various material systems through thermally- and optically-stimulated luminescence. Our outcomes highlight the transformative potential of the UCC method in advancing optical storage applications, offering significant improvements in the development of information storage solutions.

## Abstract (web summary)

Up-conversion charging (UCC) enables rapid, high-resolution optical storage in phosphors, achieving 0.01-second data writing with excellent retention and rewritability. UCC paves the way for advanced optical storage solutions.

## Graphical abstract

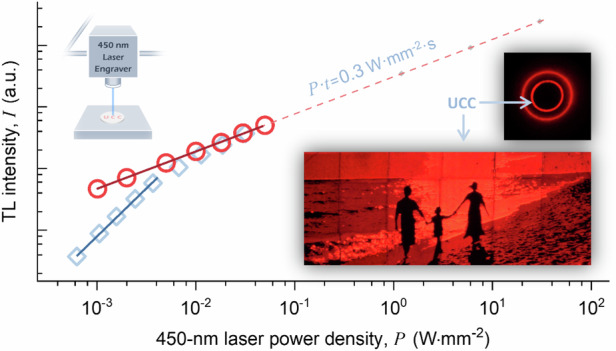

## Introduction

Optical storage is emerging as a promising alternative for information storage applications, offering significant advantages in energy-saving and data storage capacity^1^. Among the various media being explored, luminescent materials, particularly storage phosphors, are gaining attention for their role in advancing optical storage technologies^2,3^. These phosphors possess unique characteristics that allow them to capture and retain carriers upon light exposure for data recording, maintaining these charges in deep traps over extended periods^4–6^. The stability of these trapped charges is preserved unless subjected to elevated temperatures or specific light, thereby ensuring reliable and long-term information storage.

**Fig. 1:**
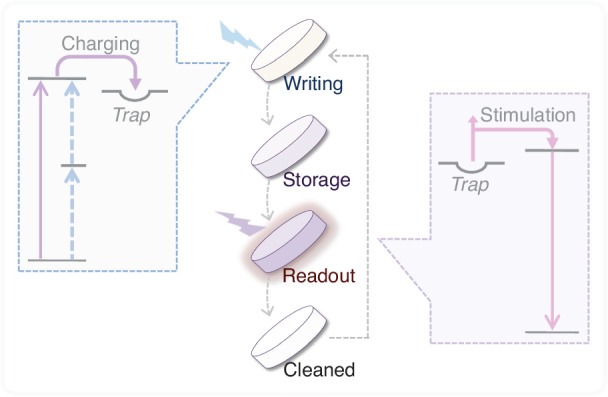
Demonstration of the optical information storage using storage phosphors. The left panel illustrates the writing mechanism, encompassing phosphor charging through optical excitation and trap filling. Beyond the conventional charging method via direct photoionization (solid arrow), an alternative approach, up-conversion charging (UCC), which involves two-step ionization (dashed arrows), can also be employed to write information into the phosphor medium. The right panel illustrates the readout mechanism, which operates on the principles of thermal- or photo-stimulated luminescence, involving the release of trapped charges and the subsequent emission from the emitting ion

Storage phosphors that exhibit thermal- or photo-stimulated luminescence are especially suited for optical data recording and retrieval^2–6^. During data recording, the phosphor medium is exposed to illumination, trapping charges in proportion to the radiation dose received. Traditionally, storage phosphors have utilized inter-band or charge-transfer transitions for direct photoionization, typically employing short-wavelength light like ultraviolet or X-rays, owing to the high-energy nature of the delocalized states within the phosphors^3–6^. This process is depicted by the solid-line arrow in the left panel of Fig. 1. The recorded information can be stored for prolonged periods and later retrieved thermally or optically as a luminescence signal^3–6^, as illustrated in the right panel of Fig. 1. This luminescence signal can then be captured by a detector, digitized, and stored on a computer. After readout, most of the trapped charges are released, and any remaining charges can be eliminated through heating or additional illumination, allowing the storage medium to be reused (Fig. 1). This feature renders storage phosphors ideal for rewritable optical memory media.

Recent research has highlighted the potential of storage phosphors involving activators such as Eu^2+^, Sm^2+^, Mn^2+^, Cr^3+^, Bi^3+^, Ce^3+^, or Pr^3+^ in optical storage applications^7–19^. Despite their promise, current technologies face challenges when it comes to efficiently recording data using visible or infrared light and achieving rapid data recording. These limitations primarily stem from traditional charging mechanisms that rely on conventional ultraviolet lamps or X-ray light as radiation sources, which typically suffer from low irradiation intensity and direct photoionization processes, leading to prolonged charging times extending to several minutes^20,21^. Consequently, the difficulties associated with high-energy excitation photons and slow charging rates have driven research toward novel strategies for more efficient optical information recording.

While extensive research on innovative methods to charge storage phosphors is lacking, recent advancements in up-conversion charging (UCC) technology present a promising solution to these challenges^22–24^. UCC utilizes a unique two-step ionization process to populate traps within phosphors, providing a non-linear excitation mechanism for charging these materials (indicated by the dashed arrows in the left panel of Fig. 1). This cutting-edge technology offers several advantages over traditional methods, including the ability to charge phosphors using visible or infrared light. It is particularly suitable for medical imaging and practical data storage applications. Encouraging outcomes have been observed in persistent phosphor systems like Zn_3_Ga_2_GeO_8_:Cr^3+^ and LiGa_5_O_8_:Cr^3+^, where UCC materials emit long-lasting light following exposure to visible or infrared illumination^22–25^. Furthermore, by utilizing the distinctive excitation and charging characteristics of UCC, this technology has demonstrated the potential to speed up charging processes and improve the overall recording efficiency of storage systems^26–28^, thereby unlocking new opportunities for storage applications.

In this study, we present a strategy for optical storage by leveraging the non-linear UCC design to enable rapid and accurate data recording in phosphor media. We demonstrate the advanced writing technique using a portable diode laser engraver with visible laser wavelengths on the Gd_3_Ga_5_O_12_:Cr^3+^ phosphor. Furthermore, we explore the applicability of UCC in various storage phosphors, highlighting its versatility in optical information storage. Realizing the full potential of UCC technology could drive substantial advancements in optical storage technology.

## Results

### Afterglow emission and up-conversion charging (UCC)

**Fig. 2:**
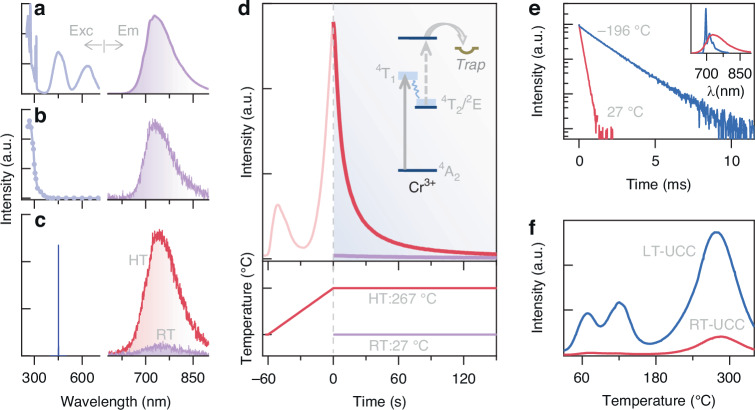
Characteristics of afterglow and up-conversion charging (UCC) in Gd_3_Ga_5_O_12_:Cr^3+^ phosphor. **a** Steady-state photoluminescence excitation and emission spectra. **b** Conventional afterglow excitation and emission spectra, with the excitation spectrum determined by measuring afterglow intensity as a function of charging wavelength and the emission spectrum obtained post-charging with 280 nm light at room temperature. **c** Afterglow emission spectra recorded at both room temperature (RT, 27 °C) and high temperature (HT, 267 °C) following a 10-s charging at room temperature with a 450 nm laser at a power density of 0.01 W·mm^−2^. The laser output (left) corresponds closely with the photoluminescence excitation band of the phosphor. **d** Temporal evolution of thermally stimulated luminescence intensities (top), with detailed experimental procedures outlined below. The inset schematically depicts the UCC process involving two-step ionization and trap-filling. **e** Fluorescence decay curves for Cr^3+^ emission upon 450 nm excitation at −196 and 27 °C, exhibiting lifetimes of 1.37 and 0.17 ms, respectively. The inset displays the corresponding photoluminescence emission spectra. **f** Thermoluminescence curves post-UCC at low temperature (LT, −196 °C) and room temperature (RT, 27 °C) following a 10-s exposure to the 450 nm laser at a power density of 0.01 W·mm^−2^

The conventional luminescence characteristics of Gd_3_Ga_5_O_12_:Cr^3+^ phosphor have been extensively studied, particularly under excitation by ultraviolet or blue light^29–32^. Upon excitation, the material exhibits steady-state photoluminescence in the deep-red spectral region at room temperature. The PL emission spectrum displays a broad band spanning 650 to 850 nm, with a peak at 720 nm (Fig. 2a). This emission transition occurs as the (Cr^3+^) ^4^T_2_ excited state transitions to the ^4^A_2_ ground state^30,31^. Analyzing this emission allows us to derive a PL excitation spectrum (left in Fig. 2a), which corresponds to the excitation transitions of Cr^3+^ ions from the ground state to the ^4^T_2_ and ^4^T_1_ levels^30,31^. Notably, the PL excitation spectrum reveals distinct lines at 311 and 276 nm, indicating energy transfer between Gd^3+^ and Cr^3+^ ions and emphasizing the involvement of the host component.

Following the cessation of ultraviolet excitation, the Gd_3_Ga_5_O_12_:Cr^3+^ phosphor exhibits long-lasting afterglow emission at room temperature (Fig. 2b). This phenomenon arises as a result of the gradual release of traps that were charged by ultraviolet irradiation, followed by the recombination of the released charge with the emitting ion^29^. When visible light excitation is employed, specifically with a filtered xenon lamp as the source, no afterglow emission occurs at room temperature. This is supported by the afterglow excitation spectrum in Fig. 2b (refer to Supplementary Fig. S1 for details). However, according to the UCC mechanism, the phosphor should be chargeable under intense visible-light illumination, especially with intense laser exposure. As predicted, exposing the Gd_3_Ga_5_O_12_:Cr^3+^ phosphor to a 450 nm laser at 0.01 W·mm^−2^ for 10 s results in a faint afterglow emission spectrum at room temperature (violet curve in Fig. 2c). Remarkably, raising the temperature significantly enhances the afterglow emission (red curve in Fig. 2c).

To investigate the temperature-dependent characteristics of UCC afterglow emission, we examined thermally stimulated luminescence and observed its changes over time. Initially, we charged the phosphor at room temperature using the 450 nm laser as mentioned above. Then, we recorded the afterglow decay at the same temperature, as shown by the violet curve in the upper section of Fig. 2d. Subsequently, we heated the phosphor at a constant rate of 4 °C·s^−1^ for 60 s until reaching 267 °C, where it was held steady. The red curves in the upper section depict the evolution of thermally stimulated luminescence, offering insights into the influence of temperature on trap distribution within the phosphor. The experimental outline is presented in the lower part of Fig. 2d. Consistent with the experimental procedure, the charged phosphor exhibited intense afterglow emission at 267 °C, followed by a gradual decay in emission intensity due to trap depletion at that temperature. Notably, the effect of thermal quenching during the thermally stimulated luminescence process in the phosphor was insignificant, as evidenced in Supplementary Fig. S2. The inset of Fig. 2d presents a schematic representation of the two-step excitation mechanism of UCC, involving the promotion of intermediate-state electrons to a high-energy delocalized state, which facilitates trap filling through a non-linear excitation pathway where the extended lifetime of the intermediate state is crucial.

To evaluate the lifetime of the intermediate state in the Gd_3_Ga_5_O_12_:Cr^3+^ phosphor, we performed fluorescence lifetime measurements at different temperatures while monitoring the Cr^3+^ emission. At room temperature (27 °C), we documented a lifetime of 0.17 ms, as presented by the red curve in Fig. 2e. Comparing this with the room temperature lifetime from the ^2^E/^4^T_2_ coupled state suggests that Cr^3+^-activated phosphors may exhibit a longer emission lifetime from the ^2^E composition at lower temperatures^32^. In line with this anticipation, measurements at liquid-nitrogen temperature (−196 °C) revealed a lifetime of 1.37 ms, represented by the blue curve in Fig. 2e. The increased lifetime of the intermediate state at lower temperatures implies a more efficient up-conversion excitation process.

To further explore the effects of irradiation temperature on the UCC process, we conducted thermoluminescence measurements to assess trap occupation in Gd_3_Ga_5_O_12_:Cr^3+^. The phosphor was exposed to a 450 nm laser at a power density of 0.01 W·mm^−2^ for 10 s at −196 and 27 °C. As shown in Fig. 2f, the thermoluminescence intensity increased nine-fold when the phosphor was charged at the lower temperature, indicating a more efficient UCC excitation. This finding suggests that the phosphor irradiated at a lower temperature will exhibit a more pronounced afterglow emission. This hypothesis was further confirmed by the afterglow emission spectra recorded at 267 °C, following UCC at −196 and 27 °C with the 450 nm laser (Supplementary Fig. S3).

### Dynamics of UCC

**Fig. 3:**
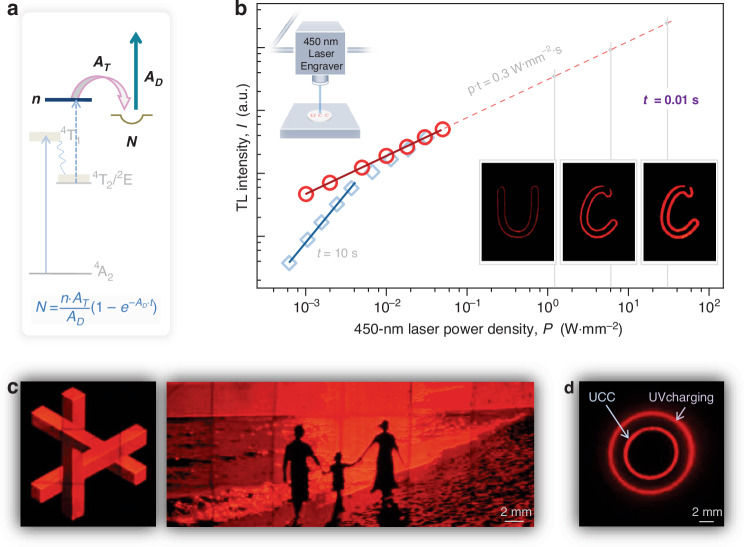
Dynamics and imaging of UCC in Gd_3_Ga_5_O_12_:Cr^3+^ phosphor. **a** Schematic of trapping and detrapping in the UCC process (see the text for further details). **b** Double-logarithmic plots depicting thermoluminescence intensities (*I*) versus power densities (*P*) of a 450 nm laser. The square curve depicts the *I*-*P* relationship measured with a fixed illumination duration of 10 s, while the circle curve illustrates the *I*-*P* under a constant dose of 0.3 W·s·mm^−2^. The upper inset provides a profile of the portable laser engraver equipped with a 450 nm diode laser module and XY-axis guide rails. The lower inset displays afterglow images captured at 267 °C following phosphor charging using the laser engraver at −196 °C. The three letters were recorded using the laser module at varying power densities with a constant 0.3 W·s·mm^−2^ dose. The illumination parameters for the three images were 1.2 W·mm^−2^ for 0.25 s, 6 W·mm^−2^ for 0.05 s, and 30 W·mm^−2^ for 0.01 s, respectively. **c** Afterglow images with stereoscopic and depth effects using UCC, recorded under the same imaging conditions as in (**b**). **d** Resolution advantage of UCC, shown with a smaller circle compared to traditional UV methods. Images taken with a 0.6-s shutter

Besides the non-linear excitation, the UCC mechanism significantly involves the electron-transfer process. A charging dynamics model, depicted in Supplementary Fig. S4, has been developed to elucidate this fundamental process^27,28^. By analyzing the UCC dynamics, it is possible to manipulate the competition between trapping and detrapping by adjusting the irradiation power and duration, as demonstrated in Supplementary Fig. S5. This manipulation provides valuable insights into the intricate processes involved. To enhance clarity, we have refined the description of UCC dynamics in Fig. 3a. Specifically, when the Gd_3_Ga_5_O_12_:Cr^3+^ phosphor is exposed to blue laser illumination, the Cr^3+^ ion undergoes a two-step excitation, leading to ionization. Ionized electrons are then captured by traps, while the excitation light simultaneously energizes some of the trapped electrons, enabling their release from the traps^27,28^. The temporal evolution of the trap population (*N*) during the charging phase is governed by the rate equation, d*N*/d*t* = *n*∙*A*_T_−*N*∙*A*_D_, where *n* represents the population in the delocalized excited state, *A*_T_ is the trap capture rate, and *A*_D_ is the photo-stimulated emptying rate of traps (see Fig. 3a). Solving this equation yields ln|*n*∙*A*_T_*-N*∙*A*_D_ | = −*A*_D_∙*t* + *C*, with *C* being an integration constant and determined by the initial conditions, *N*(*t* = 0) = 0. The trap population is then given by

1 $$N=\left(n\,\cdot\, {A}_{T}/{A}_{D}\right)\,\cdot\, \left[1-\exp \left(-{A}_{D}\,\cdot\, t\right)\right]$$

The excited state population (*n*) depends on the irradiation power (*P*) and the number of blue photons (*b*) involved, with *n* approximately proportional to *P*^*b*^. The trap emptying rate under illumination is considered roughly proportional to the irradiation power of the blue laser^28^.

Further examination of Eq. (1) demonstrates how excitation light parameters, specifically irradiation power and duration, affect the interplay between trapping and detrapping processes. In cases where the impact of the excitation light on trap emptying is minimal, a straightforward calculation involving the emptying rate and duration can be used to estimate the trap population. Applying a first-order Taylor expansion of exp(-*A*_D_∙*t*), Eq. (1) simplifies to *N* = *n·A*_T_∙*t*. Conversely, the relationship between trap occupancy and irradiation power changes when the excitation light significantly influences trap emptying, resulting in a high *A*_D_∙*t* value.

In examining UCC in Gd_3_Ga_5_O_12_:Cr^3+^ phosphor, the integrated thermoluminescence intensity (*I*), correlating with the trap population *N*, was plotted as a square curve in Fig. 3b under different irradiation power levels with a constant irradiation duration. The irradiation duration was set at 10 s, and the phosphor was charged using a 450 nm laser at liquid nitrogen temperature to prevent optical heating (Supplementary Fig. S6). Thermoluminescence curves were recorded at different power densities (Supplementary Fig. S7a). As predicted, within a valid irradiation dose range of 0.006 − 0.04 W·s·mm^−2^, the *I*-*P* curve followed a quadratic function (*I*∝*P*^1.55^). Beyond the irradiation dose of 0.04 W·s·mm^−2^ (i.e., 0.04 J·mm^−2^), the slope of the *I*-*P* curve decreased (as shown by the square curve in Fig. 3b), aligning with theoretical expectations.

In practical scenarios, such as utilizing the Gd_3_Ga_5_O_12_:Cr^3+^ phosphor for information storage under a fixed irradiation dose *P*∙*t* [keeping the term exp(-*A*_D_∙*t*) in Eq. (1) constant], the relationship between thermoluminescence intensity and irradiation power can be simplified to *I*∝*P*^*b*−1^ in Eq. (1). When exploring the UCC involving a two-step ionization at a fixed *P·t*, and in the absence of excitation saturation in the up-conversion intermediate state (Supplementary Fig. S8), we can examine the *I*-*P* relationship. To further investigate this, we conducted thermoluminescence measurements with a consistent radiation dose of 0.3 W·s·mm^−2^, which was optimized for phosphor charging performance and subsequently employed in optical storage demonstrations using the laser engraver (Supplementary Fig. S7b). Our analysis of the integrated thermoluminescence intensities (represented by the circle curve in Fig. 3b) validates the expected trend where *I* is proportional to *P* (i.e., *I*∝*P*^0.61^). This indicates that increasing the 450 nm irradiation power while maintaining the exposure dose constant results in higher thermoluminescence intensity. This specific combination, characterized by high radiation power and short exposure duration, is believed to effectively fill traps within the phosphor, as roughly predicted by the trend line in Fig. 3b. It should be noted that at very high irradiation intensities, factors such as intermediate-state saturation and ground-state bleaching may cause deviations from this prediction^33^. This improved trap filling, inversely related to irradiation duration, is a fundamental feature of UCC, stemming from its two-step ionization mechanism. In contrast, conventional direct-ionization charging with a fixed dose exhibits little dependence on either irradiation duration or power, aligning more closely with the theoretical relationship *I*∝*P*^0^ (see Supplementary Fig. S9). The improved trap-filling performance under high-power, short-duration exposure highlights a distinct advantage of the UCC method, offering potential benefits for rapid data storage applications in phosphor media and distinguishing it from conventional charging techniques.

To visually validate the UCC dynamics in Gd_3_Ga_5_O_12_:Cr^3+^, we conducted afterglow imaging experiments using a portable laser engraver equipped with a 450 nm diode laser module, as illustrated in the upper inset of Fig. 3b. Throughout the measurements, we kept the irradiation dose constant at 0.3 W·s·mm^−2^, while varying the irradiation power levels. The lower inset of Fig. 3b displays afterglow images of the letters “U”, “C” and “C”, which were written onto the phosphor surface as stored information at liquid nitrogen temperature using the laser engraver. The stored information was subsequently retrieved by heating the phosphor to 267 °C. For writing the three letters, distinct laser configurations were employed. The first utilized a power density of 1.2 W·mm^−2^ with a 0.25-s exposure per bit. The second applied a more intense 6 W·mm^−2^ for a shorter 0.05-s exposure time. Lastly, the third letter was written using the highest power density of 30 W·mm^−2^, requiring an exposure duration of only 0.01 s. The images demonstrate a progressive increase in afterglow brightness from the three letters, approximately aligning with the trend shown in Fig. 3b.

It is important to note that the portable laser engraver employed in this study is limited by its maximum laser frequency, allowing only up to 100 irradiations per second on the phosphor. Consequently, using a more advanced laser system could potentially decrease the writing time for each bit to less than 0.01 s. The fast-writing capability inherent to the UCC approach offers clear advantages for efficient data recording in optical storage applications. In addition, the variation in afterglow brightness depicted in Fig. 3b suggests that multiplexing is achievable in the Gd_3_Ga_5_O_12_:Cr^3+^ phosphor. By adjusting the power and dose levels of the 450 nm laser, we can manipulate the afterglow brightness to store optical data. Figure 3c shows two afterglow images captured under identical imaging conditions to those depicted in Fig. 3b, highlighting the stereoscopic and depth-of-field effects within UCC-afterglow imaging. The left image features three geometric building blocks, each featuring three distinct surfaces. To achieve this image, we utilized the laser engraver to write three layers of information onto the sample surface, with each layer corresponding to a specific brightness level (see Supplementary Fig. S10 for details). These brightness levels were captured following UCC at varying power densities, while maintaining a consistent illumination dose, as indicated by the laser parameters in the *I*-*P* relationship in Fig. 3b. The right image in Fig. 3c replicates a scene of three people walking along the beach. The UCC treatment similarly records three layers of information on the sample surface (Supplementary Fig. S10), resulting in the afterglow image with depth-of-field effect. These images demonstrate an enhanced storage capacity beyond simple monochromatic information, with each pattern exhibiting well-defined edges due to the high-resolution inherent in the non-linear excitation of the UCC process. To showcase the resolution advantages of the UCC technique, we conducted comparative analyses of afterglow imaging using phosphors charged by two different methods. As illustrated in Fig. 3d, the smaller circle was created using the UCC method with a 450-nm diode laser coupled to a 400 µm fiber. In comparison, the larger circle was produced using the traditional ultraviolet charging method with a 266-nm solid-state laser, also connected to the same fiber. This comparison distinctly highlights the superior resolution provided by the UCC approach. Interestingly, some storage phosphors, such as e.g., SrAl_2_O_4_:Eu^2+^,Dy^3+^, can be directly charged with blue light via single-photon absorption followed by trap filling. However, in such instances, achieving high-resolution data storage is not feasible (refer to Supplementary Fig. S11).

### Long-term storage capability and rewritability

**Fig. 4:**
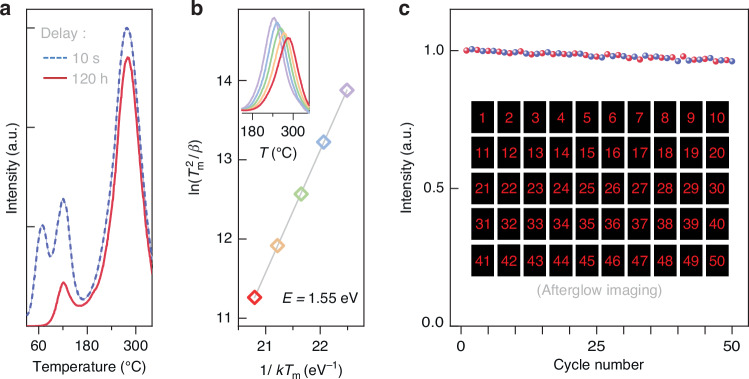
Examination of the storage capability and rewritability of the Gd_3_Ga_5_O_12_:Cr^3+^ phosphor. **a** Thermoluminescence curves acquired following UCC with a 450 nm laser at −196 °C. The dashed curve depicts data recorded after a 10-s delay, while the solid line represents data collected after a 120-h delay at room temperature. **b**, Evaluation of trap depth through thermoluminescence measurements conducted at various heating rates from 0.25 to 4 °C·s^−1^, as depicted in the inset, revealing trap depth of 1.55 eV according to Hoogenstraaten’s method. **c** Repeatability experiments involving cycles of UCC and thermal bleaching to demonstrate the reusability of the phosphor for storage purposes. The trend of afterglow intensity indicates negligible reduction in storage capacity even after 50 cycles. The inset visually illustrates this reusability, displaying afterglow images captured during each cycle. These images feature red numerical values corresponding to afterglow emissions from patterns written on the phosphor using the 450 nm laser engraver. Each red number denotes a specific instance of recycling on the same phosphor. Notably, the imaging data differs from the spectral intensity measurements, as the latter employs a uniform laser and spot size. The images were captured using a digital camera with a 0.6-s shutter speed

The observed UCC performance and subsequent afterglow emission within the Gd_3_Ga_5_O_12_:Cr^3+^ phosphor stem from the presence of traps, which play a crucial role in retaining excitation energy over extended periods. To validate the long-term storage capability, we conducted thermoluminescence measurements at different delay times, as shown in Fig. 4a. The dashed curve in Fig. 4a illustrates the thermoluminescence curve obtained immediately after ceasing 450 nm laser exposure (0.01 W·mm^−2^ for 10 s), displaying distinct bands corresponding to shallow and deep traps. As the delay time extended to 120 hours (solid-line curve in Fig. 4a), the deep trap retained approximately 85% of the initial signal, affirming its long-term retention potential.

To determine the storage duration of the deep trap in the Gd_3_Ga_5_O_12_:Cr^3+^ phosphor, we performed thermoluminescence measurements at varying heating rates, considering first-order or general-order kinetics. The inset of Fig. 4b shows that the thermoluminescence peak temperature (*T*_*m*_) shifts from 243 to 285 °C as the heating rate (*β*) increases from 0.25, 0.5, 1, 2, to 4 °C·s^−1^. Employing Hoogenstraaten’s method, we plotted ln(*T*_*m*_^*2*^/*β*) against (1/*kT*_*m*_)^34,35^, as shown in Fig. 4b. Linear fitting of this plot allowed for the determination of the trap depth (*E*), calculated to be 1.55 eV, and the frequency factor (*s*), found to be 2.08 × 10^13^ s^−1^. Subsequently, utilizing the Arrhenius equation, τ = *s*^−1^exp(*E*/*kT*), we estimated the storage time of the deep trap to be approximately 1.3 × 10^5^ years at room temperature. This calculation highlights the exceptional stability and effectiveness of the deep trap in preserving information over extended periods. However, it is essential to note that this estimated storage time represents an idealized scenario. The observed 15% reduction in deep-trap thermoluminescence intensity after 120 h (Fig. 4a) suggests the presence of competing loss mechanisms, such as tunneling or detrapping via shallower traps. Further investigation into these alternative pathways is necessary to fully understand the long-term stability of energy storage within this phosphor.

As a promising candidate for optical storage, an ideal phosphor should possess the ability to easily clear traps and refill them^1^. To demonstrate the reusability of the Gd_3_Ga_5_O_12_:Cr^3+^ phosphor for storage purposes, we conducted repeatability experiments involving cycles of UCC and thermal bleaching. Each cycle consisted of exposing the same phosphor to a 450 nm laser for 10 s with a power density of 0.01 W·mm^−2^ at liquid-nitrogen temperature, followed by 24-h storage at room temperature in the dark. Subsequently, the phosphor was heated to 267 °C to record its high-temperature afterglow emission spectrum (Supplementary Fig. S12). The phosphor was then subjected to bleaching by heating it to 400 °C to empty the traps, thereby erasing any residual data. The evolution of afterglow intensity, as depicted in Fig. 4c, showed negligible reduction in storage capacity even after 50 cycles, indicating the phosphor’s excellent reusability and robustness against laser exposure and heating in optical storage applications.

To visually illustrate the reusability of the Gd_3_Ga_5_O_12_:Cr^3+^ phosphor, we conducted a separate experiment, depicted in the inset of Fig. 4c. Using the 450 nm laser engraver, we wrote patterns onto the same phosphor sample under conditions identical to those used in the spectral recycling experiment. The resulting red numbers correspond to individual recycling instances, with each image showcasing the afterglow emission during a specific cycle. Notably, the phosphor maintained a consistent level of afterglow brightness across 50 cycles over a 50-day period. This demonstration of substantial rewritability further emphasizes the material’s versatility for passive storage applications.

### UCC in extended phosphor systems

**Fig. 5:**
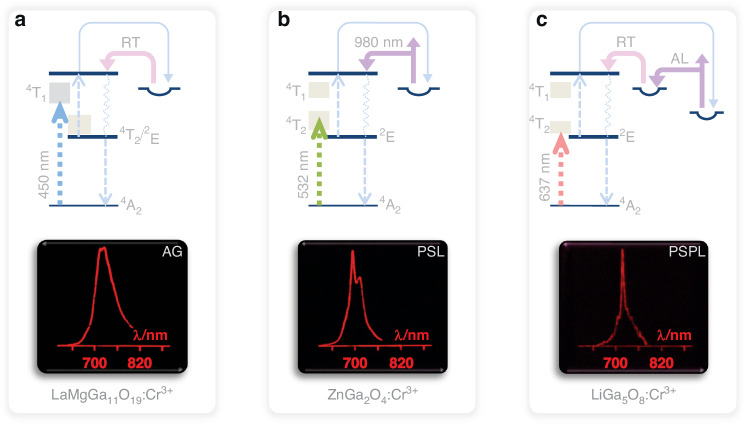
Schematic illustrations of UCC and corresponding luminescent imaging of various phosphors, when subjected to extended charging wavelengths. **a** Post-charging of LaMgGa_11_O_19_:Cr^3+^ using a 450 nm diode laser engraver, a luminescent image is generated, depicting the profile of the afterglow (AG) emission spectrum at room temperature (RT). **b** Upon charging ZnGa_2_O_4_:Cr^3+^ to the laser engraver with 532 nm laser output, a luminescent image is produced, illustrating the profile of the photo-stimulated luminescence (PSL) emission spectrum, with stimulation provided by a 980 nm fiber-coupled laser. **c** Following exposure to the laser engraver with 637 nm diode laser, the LiGa_5_O_8_:Cr^3+^ phosphor generates a luminescent image, illustrating the profile of the photo-stimulated persistent luminescence (PSPL) emission spectrum. In this instance, following a 120-h decay period, the charged phosphor is exposed to ambient light (AL), which acts as a stimulus to transfer electrons from deep to shallow traps, thereby rejuvenating the room-temperature afterglow emission. The images in (**a**–**c**) were captured using a digital camera with shutter speeds of 1 s, 10 s, and 30 s, respectively

Our research outcomes have demonstrated that the Gd_3_Ga_5_O_12_:Cr^3+^ phosphor exhibits rapid recording, stable storage, and effective retrieval capabilities when subjected to a blue laser using the UCC technique. Moreover, analysis of the excitation spectra (refer to Fig. 2a, b) suggests that red light charging is also viable, as supported by Supplementary Fig. S13. These encouraging results indicate that other gallate compositions with Cr^3+^ activation might also hold promise for optical storage purposes. To further explore this potential, we visually presented the UCC performance of various materials, including LaMgGa_11_O_19_:Cr^3+^, ZnGa_2_O_4_:Cr^3+^, and LiGa_5_O_8_:Cr^3+^ phosphors as potential candidates for optical storage media^24–26^. Prior to conducting imaging experiments, we examined the energy-level structures of the three phosphors to confirm the feasibility of UCC with extended excitation wavelengths, as depicted in Fig. 5.

In the investigation involving the LaMgGa_11_O_19_:Cr^3+^ phosphor^26^, we used a 450 nm laser for illumination at room temperature and subsequently measured the afterglow emission spectrum, as illustrated in Supplementary Fig. S14a. To visually showcase the UCC performance, we wrote the emission spectrum onto the phosphor as stored information using the 450 nm diode laser engraver, displayed in the UCC/afterglow mechanism diagram and the afterglow pattern in Fig. 5a. In the case of the ZnGa_2_O_4_:Cr^3+^ phosphor^25^, we used a 532 nm laser for charging, followed by a 12-hour dark period until the afterglow was no longer detectable. We then measured the photo-stimulated luminescence (PSL) by exposing the sample to a 980 nm fiber-coupled laser. A 790 nm short-pass filter (Semrock, TSP01-790) was employed to block the stimulating light, with the resulting PSL emission spectrum shown in Supplementary Fig. S14b. For imaging purposes, this spectrum was written onto the phosphor as stored information using the laser engraver equipped with a 532 nm laser module, followed by reading out as the PSL signal, consistent with the spectral measurement procedure. The UCC/PSL mechanism diagram and the luminescent image are presented in Fig. 5b.

For the LiGa_5_O_8_:Cr^3+^ phosphor, UCC was achieved with a 637 nm laser, and we observed the phenomenon of photo-stimulated persistent luminescence (PSPL) at room temperature^24^. During the PSPL process, electrons in deep traps were stimulated by ambient light, moving to shallow traps and rejuvenating the afterglow signal for data retrieval. To record the PSPL emission spectrum, we kept the charged phosphor in darkness for 120 hours until no afterglow was observable. Subsequently, the phosphor was exposed to indoor ambient light (85-W Philips LED ceiling lamp) for 60 s, after which the PSPL emission spectrum was measured, as presented in Supplementary Fig. S14c. For imaging, the emission spectrum was written onto the phosphor using the diode laser engraver with a 637 nm laser module, followed by reading out as the PSPL signal, in alignment with the spectral measurement procedure. The UCC/PSPL mechanism diagram and the corresponding luminescent image are outlined in Fig. 5c. Notably, the laser engraver used for pattern creation maintained consistent power densities of 30 W·mm^−2^ and exposure times for 0.01 s across all three lasers. This short irradiation duration offers the advantage of minimizing the impact of optical heating on trap filling. The present findings highlight the potential of Cr^3+^-activated gallate phosphors in advancing programmable information storage.

While Cr^3+^-doped phosphors have shown promise for UCC-based optical storage, some other lanthanide and transition metal ions like Pr^3+^, Tb^3+^, or Mn^2+^ could offer alternative avenues for this technology^27,36,37^. To validate this, we examined a Y_3_Al_2_Ga_3_O_12_:Pr^3+^ garnet composition, known for its ultraviolet emission^38^, as a potential UCC material. Information was written onto the phosphor using the 450 nm laser engraver. Significantly, a clear ultraviolet signal might be recovered during readout in indoor lighting conditions (Supplementary Fig. S15). This finding suggests that UCC-based optical storage, using appropriately designed materials, could function effectively even in brightly lit environments.

## Discussion

Our study demonstrates the exciting potential of the up-conversion charging (UCC) approach for efficient optical storage in phosphors. By exploiting the non-linear excitation/charging properties and employing a portable laser engraver with visible diode lasers, we have effectively integrated the UCC technique with the storage capabilities of a Cr^3+^-activated gallate composition, Gd_3_Ga_5_O_12_:Cr^3+^, achieving precise data recording with each bit requiring only 0.01 s of exposure. The inherent trap distribution within the phosphor facilitates long-term data preservation, with distinctive afterglow patterns retrievable through heating or illumination. These outcomes highlight the considerable promise of UCC technology in advancing optical storage systems, marking a substantial advancement in data storage solutions.

Despite these encouraging findings, several challenges remain. Future research efforts should aim to improve the efficiency of phosphor materials and optimize writing methodologies to boost system performance. For example, given the two-step excitation mechanism of UCC, developing transparent bulk phosphors for use as storage media is a valuable goal, potentially enabling three-dimensional optical data storage and expanding the scope of this technology^39–42^. Moreover, UCC has the potential to further improve writing resolution by enabling spatially super-resolved optical storage with phosphors through the two-step ionization process, wherein data is inscribed at the intersection of two excitation beams. This approach can surpass the limitations of traditional linear optics, such as Abbe’s diffraction limit^42–45^. The continuous evolution of UCC technology holds the potential to revolutionize data storage, unlocking new possibilities in various scientific and technological domains.

## Materials and methods

### Material synthesis

Gallate phosphors were synthesized for optical storage media via a solid-state synthesis method. The Gd_3_Ga_4.995_Cr_0.005_O_12_ composition (referred to as Gd_3_Ga_5_O_12_:Cr^3+^ for simplicity) was prepared by mixing Gd_2_O_3_, Ga_2_O_3_, and Cr_2_O_3_ powders, followed by grinding and sintering at 1500 °C for 3 h in the air. Similar procedures were employed for synthesizing LaMgGa_11_O_19_:Cr^3+^, ZnGa_2_O_4_:Cr^3+^, and LiGa_5_O_8_:Cr^3+^ compositions.

### Luminescence measurements

Steady-state photoluminescence (PL) and afterglow measurements were performed using a CCD spectrometer (Ocean Optics, QEPro). The PL excitation spectrum was obtained by illuminating the sample with a filtered xenon lamp (Energetiq, EQ-99x-FC) and recording the emission intensity. For afterglow excitation measurements, samples were exposed to specific xenon light wavelengths from 260 to 700 nm for 60 s. After the cessation of excitation, the afterglow emission intensity was recorded after a delay of 60 s. In the spectral measurements, excitation sources included the xenon lamp and a 450 nm laser diode (CNI, MDL-XD-450). Excitation power was monitored using an optical power meter (Newport, 2936-R). Thermoluminescence curves were generated using a TL Reader (Rongfan, SL08 improved version) at a heating rate of 4 °C·s^−1^. Before each measurement, the phosphor was heated at 400 °C twice to eliminate any residual storage traps.

### Afterglow imaging

Imaging experiments were conducted utilizing the UCC method, with images captured by a SONY 7s2 (ILCE-7SM2) camera. Programmable data was stored on the phosphors using a portable diode laser engraver, which allowed precise control over the charging process. This laser engraver was a commercially available model (DAJA, D2-20W-OME) featuring a primary laser wavelength of 450 nm and an adjustable power output up to 20 W. It has a beam area of 0.1 mm^2^, with dimensions of 0.5 mm in width and 0.2 mm in height. The device supports scanning speeds from 0.01 to 1 s per bit. Besides the 450 nm laser module, 532 and 637 nm lasers compatible with pulse width modulation (PWM) and equipped with 3-pin terminal connections are integrated. To optimize the engraver for the UCC technique, we operated the control software to enable precise adjustments of both laser power and exposure time. This precision enabled us to charge the phosphor in a customized design, resulting in detailed and desired information being written on the surface of the phosphor without causing any damage or degradation. It is significant to mention that the current recording resolution was limited by the size of the laser beam, while the storage speed was dependent on the capabilities of the present machine, which had a maximum laser output frequency of 100 times per second.

## Supplementary information

Supplementary Information

## Data Availability

All the data supporting the findings of this study are presented within the article and its Supplementary Information. Additional data related to this paper are available from the corresponding authors upon request. Source data are provided within this paper.
