# Supplementary material for: Unlocking the potential of up-conversion charging for rapid and high-resolution optical storage with phosphors
Source: Light Sci Appl. 2025 Mar 4;14:107. doi: 10.1038/s41377-025-01746-9 (PMC11880567; doi:10.1038/s41377-025-01746-9)
Supplement: Supplementary file 1 — Supplementary Information [file 41377_2025_1746_MOESM1_ESM.pdf]

Supplementary Information

Unlocking the potential of up-conversion charging for rapid and high-resolution optical storage with phosphors

Lu Chen[1†], Xueqing Liu[1†], Feng Liu[1]*, Chuan Liao[2], Liangliang Zhang[2], Jiahua Zhang[2], Xiao-jun Wang[3]*, Yichun Liu[1]*

[1]Key Laboratory for UV-Emitting Materials and Technology of Ministry of Education, Northeast Normal University, Changchun 130024, P. R. China. [2]State Key Laboratory of Luminescence and Applications, Changchun Institute of Optics, Fine Mechanics and Physics, Chinese Academy of Sciences, Changchun 130023, P. R. China. [3]Department of Physics, Georgia Southern University, Statesboro, GA 30460, USA.

*Corresponding authors. E-mail: fengliu@nenu.edu.cn (F.L.); xwang@georgiasouthern.edu (X.W.); ycliu@nenu.edu.cn (Y.L.)

†These authors contributed equally to this work.

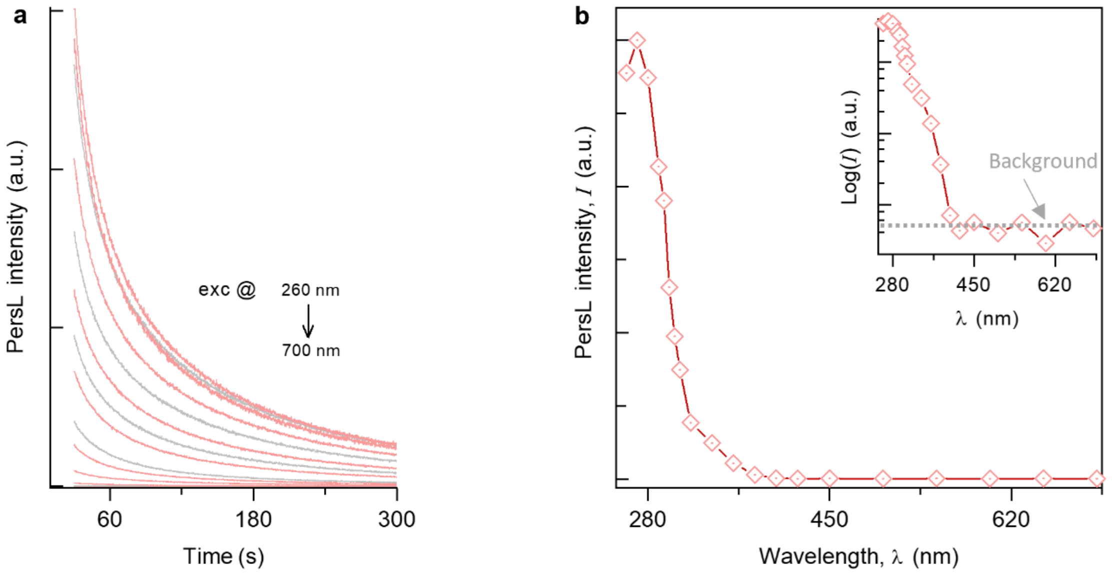

Figure S1. Excitability of the afterglow in the $Gd_3Ga_5O_{12}:Cr^{3+}$ phosphor. **a,** Afterglow decay curves measured following exposure to xenon light filtered across a wavelength range of 260 to 700 nm. **b,** Afterglow excitation spectrum obtained by associating afterglow decay intensities with the corresponding wavelengths of the illuminating light. This spectrum corresponds to the one depicted in Fig. 2b. The inset displays the same excitation spectrum on a logarithmic intensity scale, demonstrating that wavelengths between 400 and 700 nm of xenon light are ineffective in creating the afterglow.

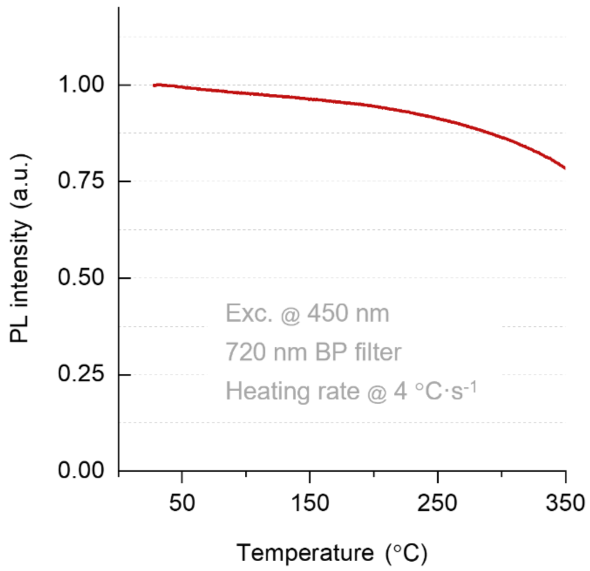

Figure S2. Steady-state photoluminescence (PL) emission intensity as a function of temperature for $Gd_3Ga_5O_{12}:Cr^{3+}$ phosphor, excited at 450 nm with a power density of 0.1 $mW·cm^{-2}$. The emission intensity was monitored through a 720 nm band-pass filter (10 nm bandwidth) while the temperature was gradually raised at 4 °C/s.

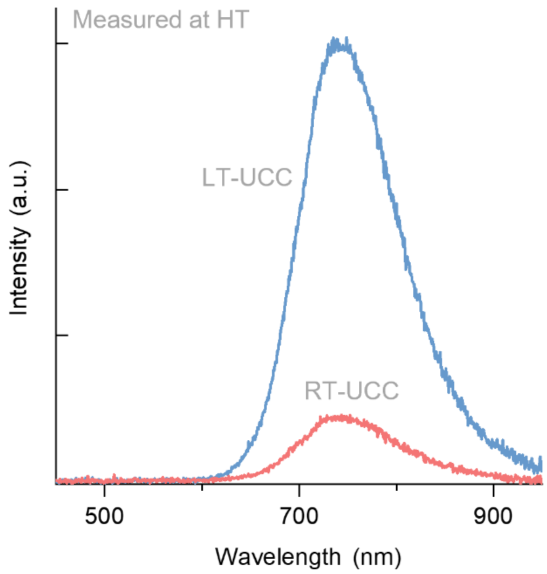

Figure S3. Afterglow emission spectra of $Gd_3Ga_5O_{12}:Cr^{3+}$ phosphor measured at high temperature (HT, 267 °C). Prior to recording the emission, the phosphor was subjected to UCC with a 450 nm laser at low temperature (LT, -196 °C) and room temperature (RT, 27 °C), respectively. The UCC protocol employed an exposure duration of 10 seconds with the laser operating at a power density of 0.01 $W \cdot mm^{-2}$. The spectrum with lower intensity is the same as the red curve in Fig. 2c.

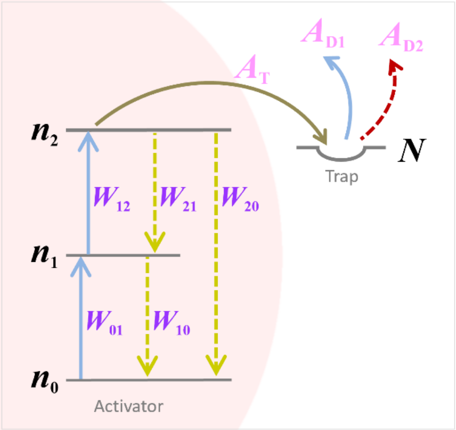

Figure S4. Schematic illustration depicting the UCC dynamics. In this representation, the variables n_0, n_1, and n_2 denote the populations of the ground state, an intermediate state, and the delocalized excited state, respectively, while N indicates the trap population. W_{xy} denotes the transition rate from state x to state y (including both radiative and non-radiative transitions), while A_T, A_{D1}, and A_{D2} represent the rates for the trapping process and the detrapping linked to excitation-light stimulation and ambient-temperature stimulation, respectively.

To depict the dynamics of up-conversion charging (*UCC*), *we present a concise schematic illustration. Our focus is on a two-photon excited-state absorption scheme, which aligns with insights gained from an energy transfer up-conversion framework. In UCC phosphor systems, traps can become populated following up-conversion excitation and electron delocalization. Simultaneously, these trapped electrons may be released via illumination energy* (*excitation-light-stimulated detrapping*) *or thermal energy* (*ambient-temperature-stimulated detrapping*). *For simplicity, we will not consider re-trapping during UCC or tunneling detrapping.*

During the illumination phase, the rate equations of state populations are as follows:

$$\frac{dn_0}{dt} = W_{10} \cdot n_1 + W_{20} \cdot n_2 - W_{01} \cdot n_0 \,, \tag{1}$$

$$\frac{dn_1}{dt} = W_{01} \cdot n_0 + W_{21} \cdot n_2 - (W_{12} + W_{10}) \cdot n_1 \,, \tag{2}$$

$$\frac{dn_2}{dt} = W_{12} \cdot n_1 + (A_{D1} + A_{D2}) \cdot N - (W_{21} + W_{20} + A_T) \cdot n_2 \,, \tag{3}$$

$$\frac{dN}{dt} = A_T \cdot n_2 - (A_{D1} + A_{D2}) \cdot N \,. \tag{4}$$

Where the meanings of these physical quantities are described in the figure caption.

A. Population in the delocalized excited state (Ref. S1)

Given that the trap-filling and -emptying processes are generally significantly slower than transitions within the activator ion, we will assume that the activator ion maintains a steady-state excited condition during UCC when exposed to a power level P. Thus, we will disregard the terms A_T, A_{D1} *and* A_{D2} *from equation* (3), *and set equations* (2) *and* (3) *to zero. Furthermore, considering the substantial population of the ground state, any variations in its population can be regarded as negligible, allowing us to treat* n_0 *as a constant. This simplification significantly reduces the complexity of equation* (1). *The population of the delocalized excited state can be expressed as follows:*

$$n_2 = \frac{W_{01} \cdot W_{12} \cdot n_0}{(W_{12}+W_{10}) \cdot W_{20} + W_{21} \cdot W_{10}} \propto \frac{n_0 \cdot P^2}{(P+W_{10}) \cdot W_{20} + W_{21} \cdot W_{10}} \,. \tag{5}$$

When the influence of W_{10} is significantly greater than P, equation (5) *simplifies to* $n_2 \propto \frac{n_0 \cdot P^2}{(W_{20}+W_{21})\cdot W_{10}}$, *indicating that n_2 is proportional to P^2. This proportionality is often used as a rule of thumb for evaluating the contribution of multi-photon excitation in the up-conversion process.*

Conversely, if the effect of W_{10} is much weaker than P, equation (5) *suggests that* $n_2 \propto (n_0 \cdot P/W_{20})$, *indicating that n_2 becomes proportional to P. In this case, the effects of nonlinear excitation lessen due to the saturation of intermediate states under high-power excitation.*

B. Trap population (Ref. S2)

By applying the method of separation of variables to equation (4), *we arrive at the following integral* $\int_{N_0}^{N} \frac{1}{A_T \cdot n_2 - (A_{D1}+A_{D2})\cdot N} dN = \int_0^t dt$, *where N_0 represents the initial trap population prior to charging, and t denotes the duration of the illumination. Solving this equation yields* $\left|\frac{A_T \cdot n_2 - (A_{D1}+A_{D2})\cdot N}{A_T \cdot n_2 - (A_{D1}+A_{D2})\cdot N_0}\right| = exp[-(A_{D1}+A_{D2})\cdot t]$. *Thus, the general solution is*

$$N = \frac{A_T \cdot n_2}{A_{D1}+A_{D2}}\left\{1 \mp \left[1 - \frac{(A_{D1}+A_{D2})\cdot N_0}{A_T \cdot n_2}\right] exp[-(A_{D1}+A_{D2})\cdot t]\right\}. \quad (6)$$

For simplicity, we first assume that the storage phosphor is a fully annealed sample, meaning that the initial traps are empty before charging (*i.e.,* N_0=0). *When the "∓ sign" in equation* (6) *is taken as negative, we derive*

$$N = \frac{A_T \cdot n_2}{A_{D1}+A_{D2}}\{1 - \exp[-(A_{D1}+A_{D2})\cdot t]\}. \quad (7)$$

Now, we will discuss the trap population under varying levels of illumination dosage.

(B.1) Low-dose illumination. *In cases where the illumination duration is short, the exponential terms $A_{D1}\cdot t$ and $A_{D2}\cdot t$ in equation* (7) *become negligible. Consequently, it is justifiable to apply the first-order Taylor series approximation to the exponential function in equation* (7), *which simplifies to* $\exp[-(A_{D1}+A_{D2})\cdot t] \approx 1-(A_{D1}+A_{D2})\cdot t$. *This allows equation* (7) *to be reformulated as* $N \propto n_2 \cdot A_T \cdot t$.

Aligning with the considerations outlined in equation (5), *the population of the delocalized excited state* (n_2) *relies on both the illumination power (P) and the number of excitation photons* (*b*), *resulting in* $n_2 \propto P^b$ *(b=2 for two-photon excitation). For the detrapping process, we can assume that* $A_{D1}\cdot t \propto P\cdot t$, *while $A_{D2}\cdot t$ remains a minor constant that can be overlooked at a given temperature. With a fixed illumination duration* (*t*), *we discover that*

$$N \propto P^b. \quad (8)$$

Alternatively, when the illumination dose $P\cdot t$ remains constant, we have

$$N \propto P^{b-1}. \quad (9)$$

(B.2) High-dose illumination. *In situations of high illumination dosage, the exponential function in equation* (7) *diminishes toward zero, leading to the relation* $N \propto P^{b-1}$. *It is crucial to understand that at extremely high illumination power, we need to factor in the discussion from equation* (5) *and substitute* $n_2 \propto (n_0 \cdot P/W_{20})$ *into equation* (7), *resulting in* $N \propto A_T$, *which indicates that the traps become filled.*

The above-mentioned scenario assumes that the initial traps were empty before charging. However, if the traps in the phosphor have been partially filled before charging, the expression $A_T \cdot n_2 - (A_{D1}+A_{D2})\cdot N_0$ *may be less than zero. In such cases, along with the continuous filling of traps, equation* (7) *still applies. Nevertheless, when the traps are partially filled, but the overall influence of illumination tends to lead to a gradual depletion of the trap, the "∓ sign" in equation* (6) *might turn out to be positive. At this point, discussions related to the trap population will become increasingly complex in this context (please refer to Ref. S2 for further details).*

References

[S1] *Pollnau, M. et al. Phys. Rev. B* ***61****, 3337* (*2000*).

[S2] *Yan, S. Y., et al. Phys. Rev. Appl.* ***13****, 044051* (*2020*).

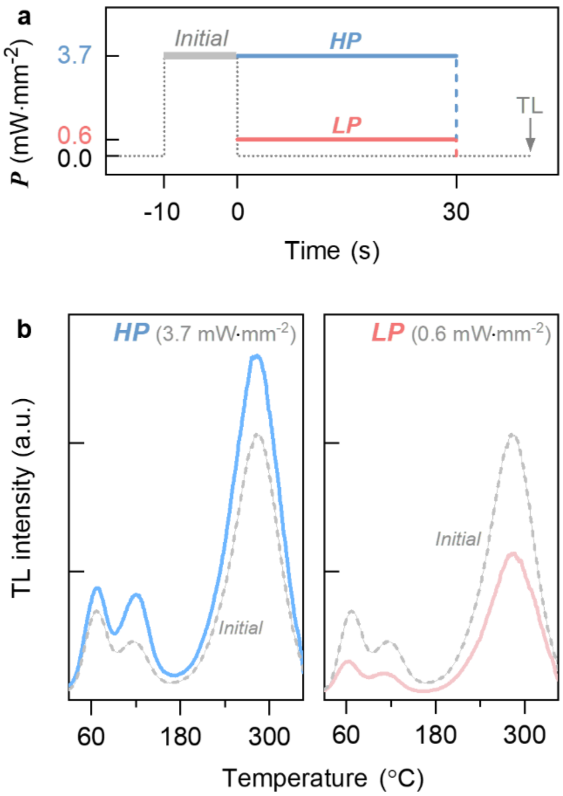

Figure S5. Competition between trapping and detrapping in $Gd_3Ga_5O_{12}$:Cr^{3+} phosphor under modulated irradiation with a 450 nm laser. **a,** Experimental protocol: The phosphor is initially exposed to the laser at a power density of 3.7 mW·mm^{-2} for 10 seconds to fill some traps (*Initial* illumination mode). Subsequently, secondary laser illuminations are conducted at 3.7 mW·mm^{-2} (high power, *HP* mode) and 0.6 mW·mm^{-2} (low power, *LP* mode) for 30 seconds each, with thermoluminescence (TL) measurements initiated at 45 seconds. **b,** Corresponding TL curves obtained following laser exposure under different illumination conditions.

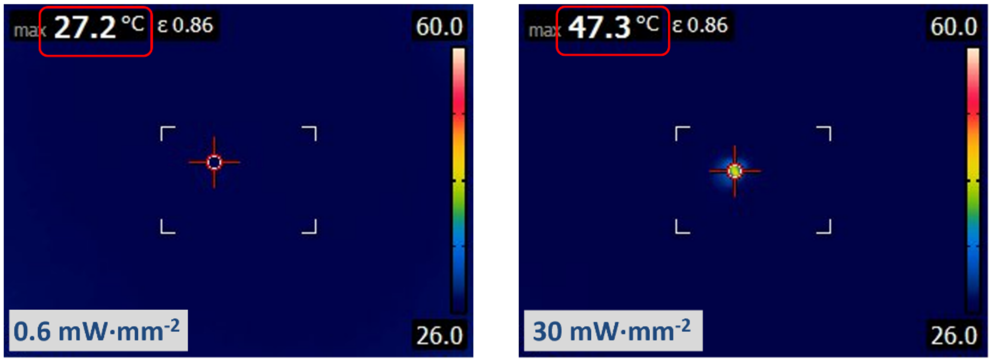

Figure S6. Evaluation of optical heating effects on the surface of $Gd_3Ga_5O_{12}:Cr^{3+}$ phosphor when exposed to the 450 nm laser at power densities of 0.6 and 30 mW·mm^{-2}. During laser exposure, temperature changes on the phosphor's surface were monitored using a thermal camera (FLIR, ETS320). The images reveal that a 10-second exposure to low-power illumination results in negligible temperature variations, whereas the high-power laser exposure for 10 seconds significantly elevates the surface temperature to at least 47°C, as resolved by the thermal camera.

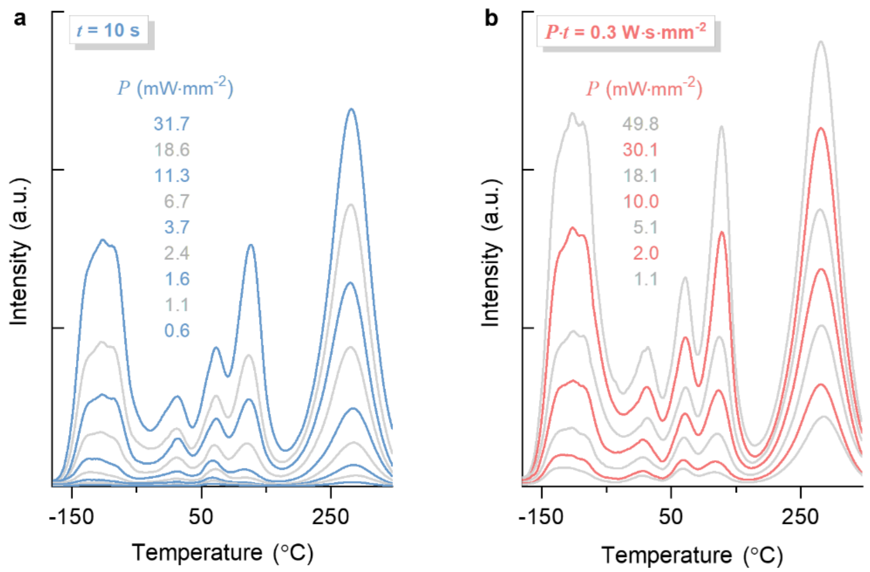

Figure S7. Thermoluminescence (TL) measurements of the $Gd_3Ga_5O_{12}$:Cr^{3+} phosphor following exposure to a 450 nm laser at -196 °C, under varying irradiation power densities while maintaining a consistent exposure duration or dose. **a,** TL curves obtained after a fixed exposure time of 10 seconds. **b,** TL curves recorded following irradiation with a constant dose of 0.3 W·s·mm^{-2}. Multiple TL peaks are evident in all curves, indicating multiple traps within the phosphor. As irradiation power changes, individual TL peaks show inconsistent intensity variations, implying that the UCC performance is not uniform across the various traps. This phenomenon likely arises from competitive trapping and detrapping processes during UCC. While irradiation power influences the TL spectral profile, the present study focuses on the integrated TL intensity, reflecting the total population of traps, to analyze the UCC behavior of the phosphor.

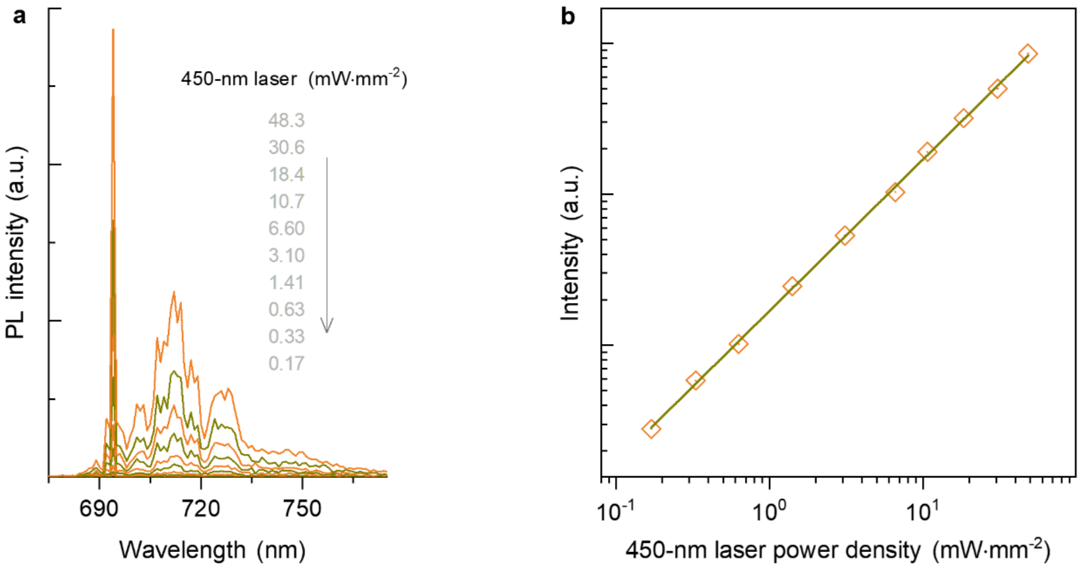

Figure S8. Examination of the intermediate-state saturation effect on the up-conversion excitation process in $Gd_3Ga_5O_{12}$:0.5%Cr^{3+} phosphor under 450 nm laser irradiation at -196 °C. **a,** Steady-state photoluminescence (PL) emission spectra acquired with varying laser power densities. **b,** PL emission intensity as a function of excitation power, demonstrating a strong correlation with a linear fit. In the phosphor, the emitting state serves as the intermediate state in the UCC excitation process, and the observed linearity suggests that intermediate-state saturation has a negligible impact within the examined power range.

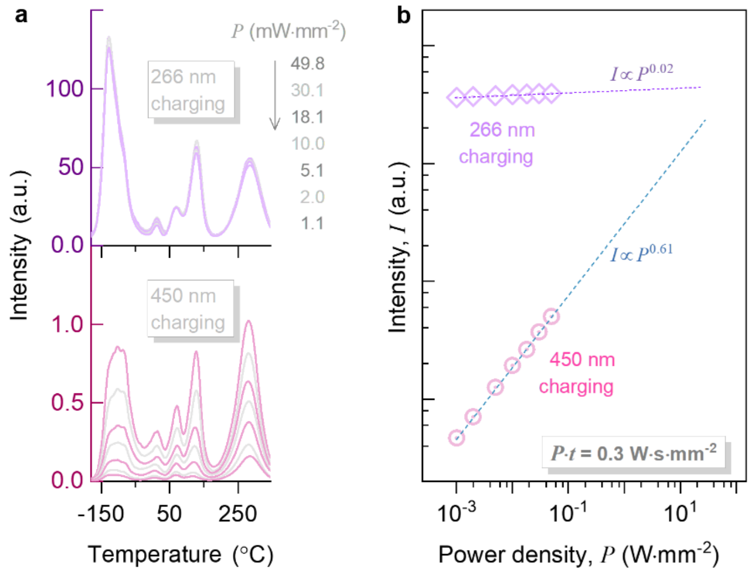

Figure S9. Effects of trapping and detrapping on trap filling in $Gd_3Ga_5O_{12}$:Cr^{3+} phosphor under 266 nm and 450 nm laser irradiation at -196 °C, with each maintaining a constant exposure dose of 0.3 W·s·mm^{-2}. **a,** Thermoluminescence curves recorded after exposure to both lasers at varying power densities, with the constant illumination dose. **b,** Double-logarithmic plots showing the correlations between thermoluminescence intensities (I) and irradiation power densities (P).

In this demonstration, UCC with a fixed dose shows a direct proportional relationship between I and P, reflective of the intrinsic characteristic of UCC due to its two-step ionization process. That is, the UCC approach allows higher power to shorten charging times and enhance trap filling. Conversely, ultraviolet charging with a constant dose exhibits power-independent thermoluminescence intensity, aligning with the single-photon absorption mechanism, where I is approximately proportional to P^0.

From the perspective of optical storage applications, while ultraviolet irradiation can facilitate rapid charging, it requires either extended periods of low-intensity exposure or short pulse of high-intensity light. The former hinders rapid data writing, while the latter proves impractical due to the absence of commercially available ultraviolet laser engravers matching the power and portability of their blue counterparts. In contrast, UCC leverages readily available blue laser technology, and its inherent requirement for high-power nonlinear excitation, facilitates rapid writing speeds.

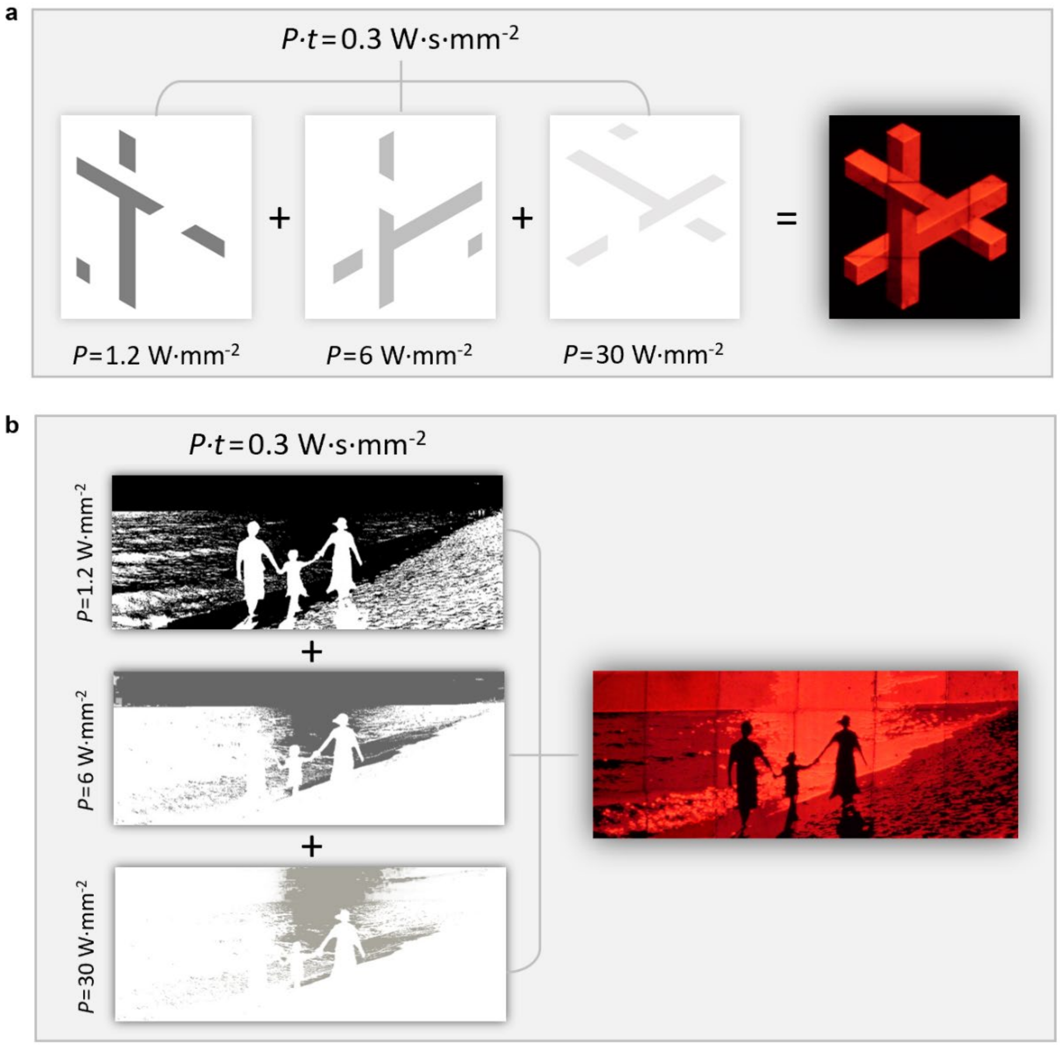

Figure S10. Multi-layered afterglow imaging utilizing $Gd_3Ga_5O_{12}:Cr^{3+}$ phosphor via UCC treatments. The images were produced using a portable laser engraver equipped with a 450 nm diode laser, delivering a fixed dose of 0.3 W·s·mm^{-2}. **a**, Geometric patterns illustrating the capability to achieve distinct afterglow intensities through modulation of laser power density. Three layers of patterns were inscribed with varying laser power densities to modulate brightness levels. **b**, Demonstration of intricate afterglow imaging depicting a scene with three individuals on a beach. Similar to pattern **a**, three layers of information were encoded onto the phosphor surface, achieving varying brightness levels while maintaining a consistent laser illumination dose.

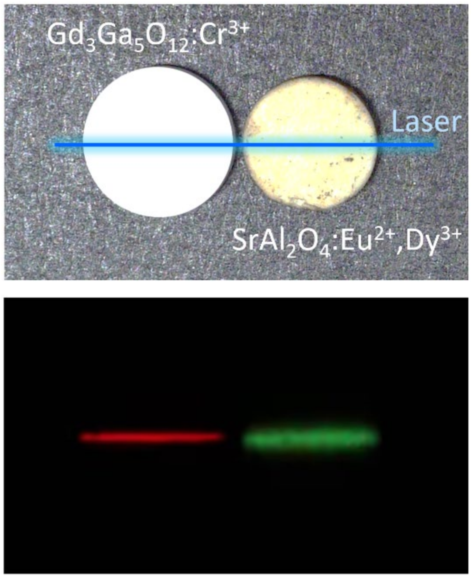

Figure S11. Demonstration of writing-in resolution using UCC and direct charging techniques through afterglow imaging. A 450 nm blue laser, at a power density of 0.01 W·mm^{-2}, was scanned across $Gd_3Ga_5O_{12}$:Cr^{3+} and $SrAl_2O_4$:Eu^{2+},Dy^{3+} discs. Post-exposure, distinct linear patterns emerged on both materials. The $Gd_3Ga_5O_{12}$:Cr^{3+} exhibited a sharp afterglow line, attributed to the two-step ionization process inherent to UCC. In contrast, the $SrAl_2O_4$:Eu^{2+},Dy^{3+} displayed a broadened afterglow line, indicative of direct charging induced by the 450 nm laser excitation.

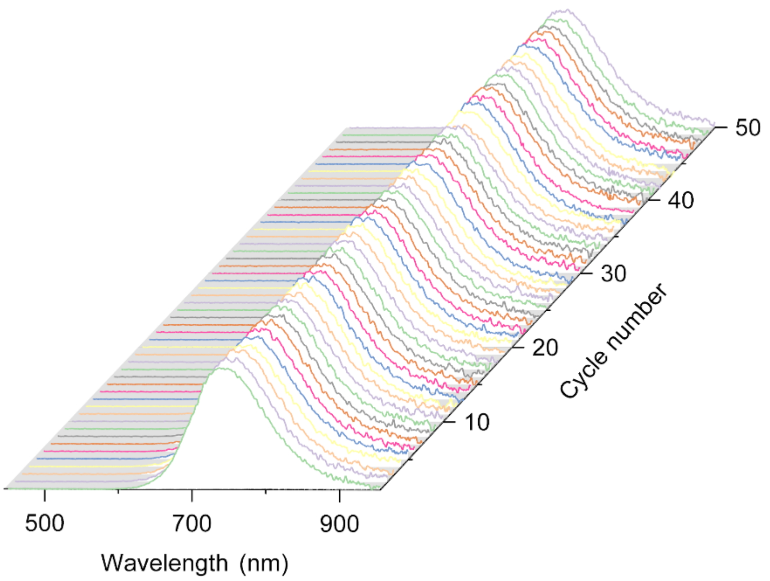

Figure S12. Assessment of the reusability of $Gd_3Ga_5O_{12}:Cr^{3+}$ phosphor under conditions of 450 nm laser exposure and thermal treatment, involving repeated cycles of UCC and thermal bleaching. The afterglow intensity trend demonstrates negligible reduction in storage capacity even after 50 cycles.

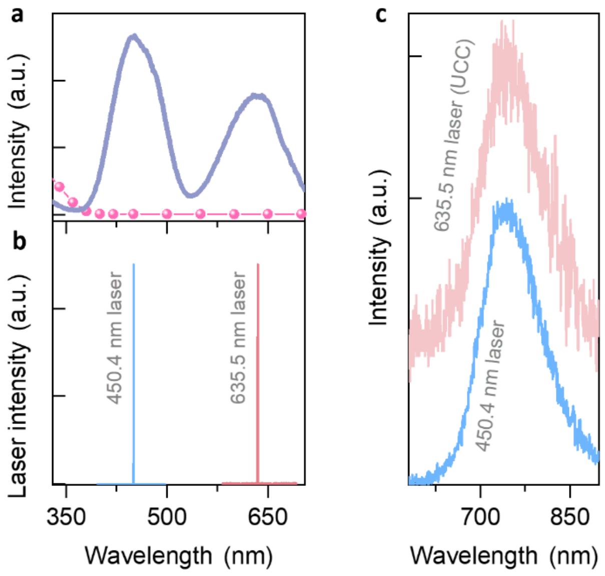

Figure S13. UCC of $Gd_3Ga_5O_{12}:Cr^{3+}$ phosphor following exposure to blue and red lasers at liquid nitrogen temperature. **a,** Excitation spectra for steady-state photoluminescence (solid line) and afterglow (ball line). **b,** Output spectra from the lasers, including a blue laser (0.01 W·mm^{-2}, with a peak at 450.4 nm) and a red laser (0.005 W·mm^{-2}, peaking at 635.5 nm). **c,** Afterglow emission spectra recorded at room temperature following a 10-second exposure to both lasers.

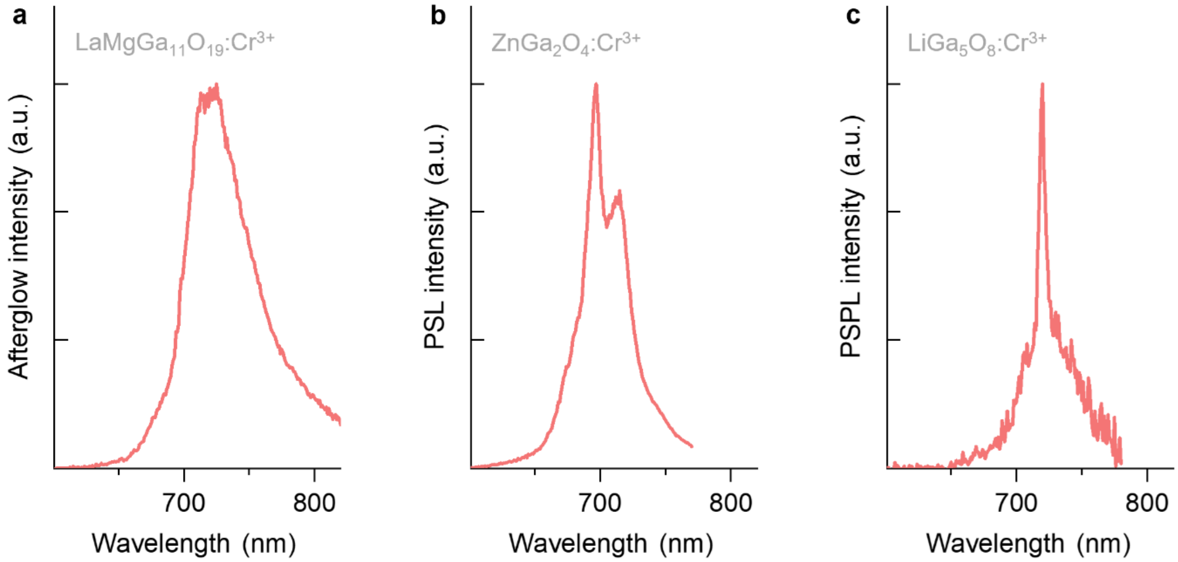

Figure S14. Emission spectra of various phosphors following UCC at room temperature, recorded using different excitation wavelengths. **a,** Afterglow emission of $LaMgGa_{11}O_{19}:Cr^{3+}$ phosphor, recorded following charging with a 450 nm laser (0.01 W·mm^{-2}, 10 seconds exposure). **b,** Photo-stimulated luminescence (PSL) spectrum of $ZnGa_2O_4:Cr^{3+}$ phosphor, obtained after charging with a 532 nm laser (0.01 W·mm^{-2}, 10 seconds) and subsequent stimulation using a 980 nm fiber-coupled laser. **c,** Photo-stimulated persistent luminescence (PSPL) of $LiGa_5O_8:Cr^{3+}$ phosphor, measured after charging with a 637 nm laser (0.01 W·mm^{-2}, 10 seconds) followed by ambient-light stimulation for 60 seconds.

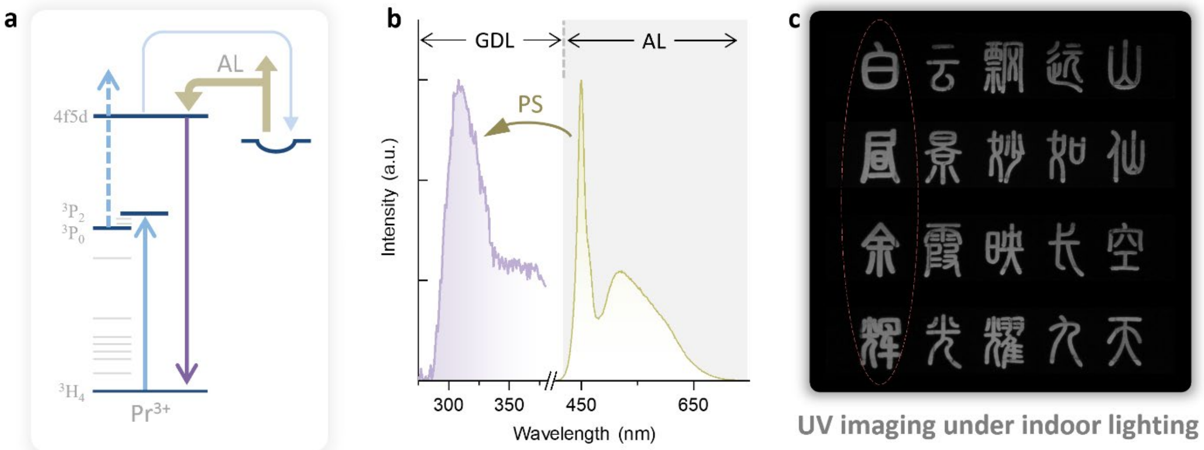

Figure S15. Achievement of UCC-based optical storage using $Y_3Al_2Ga_3O_{12}:Pr^{3+}$ phosphor. **a**, Schematic representation for UCC and the associated photo-stimulated luminescence under ambient lighting (*AL*) conditions. **b**, Glow-in-the-daylight emission spectrum, recorded after exposure to a 450 nm laser (power density, 0.01 W·mm^{-2}) at room temperature. After a 12-hour decay period, the charged phosphor is exposed to indoor *AL* (white-LED lamps from OPPLE, MJ1175-D16Z-T-01). Consequently, the phosphor emits ultraviolet wavelengths that are distinct from general *AL*. **c**, Ultraviolet afterglow imaging demonstration showcasing a Chinese poem, with each character glowing ultraviolet under *AL*. The image was generated using a portable 450 nm laser engraver, delivering a power density of 30 W·mm^{-2} with a fixed dose of 0.3 W·s·mm^{-2}. The initial characters of each line combine to form the phrase "*glow in the daylight*" in Chinese. The ultraviolet image was captured using a Touptek IUA1300KMA camera with a Hoya U-340 UV band-pass filter to detect the ultraviolet signal selectively.
